# Clinical Characterization of Korean Patients with Pseudoxanthoma Elasticum and Angioid Streaks

**DOI:** 10.3390/genes12081207

**Published:** 2021-08-04

**Authors:** Ki Won Jin, Kwangsic Joo, Se Joon Woo

**Affiliations:** Department of Ophthalmology, Seoul National University College of Medicine, Seoul National University Bundang Hospital, Seongnam 13620, Korea; realkiwon@gmail.com (K.W.J.); joo_man@hanmail.net (K.J.)

**Keywords:** *ABCC6*, angioid streaks, pseudoxanthoma elasticum, Korean population

## Abstract

This study aimed to characterize Korean patients with pseudoxanthoma elasticum (PXE) presenting with angioid streaks. Retinal phenotypes were longitudinally evaluated by multimodal ophthalmic imaging, and targeted gene panel sequencing for inherited retinal diseases was conducted. Seven subjects from unrelated families (median age, 51.2 years) were enrolled and followed for a median of 3.2 years. Four asymptomatic patients were significantly younger than three symptomatic patients with decreased visual acuity at presentation (mean age; 38.1 vs. 61.5 years, *p* = 0.020). The asymptomatic patients maintained good vision (20/32 or better) and had no choroidal neovascularization (CNV) over the observation period. The symptomatic patients showed additional reduction in visual acuity and bilateral CNV occurrence during the longitudinal follow-up. Pathogenic *ABCC6* variants were identified in all patients, leading to a diagnosis of PXE. Heterozygous monoallelic variants were identified in four patients and compound heterozygous variants were detected in three patients. Nine *ABCC6* variants were identified, including one novel variant, c.2035G>T [p.Glu679Ter]. This is the first genetic study of Korean patients with PXE.

## 1. Introduction

Angioid streaks (AS) are breaks in the Bruch’s membrane radiating from the optic disc [1,2,3]. AS area hallmark of pseudoxanthoma elasticum (PXE; OMIM #264800), and of various systemic conditions such as Paget’s disease of bone and sickle-cell anemia [4]. AS can be associated with choroidal neovascularization (CNV) [4].

PXE is an autosomal recessive hereditary disease that causes multi-systemic ectopic mineralization of elastic fibers most apparent in the skin, eye, and blood vessels [5]. PXE is caused by a loss-of-function mutation in the ATP-binding cassette subfamily C member 6 (*ABCC6*) gene (ENSG00000091262) located on chromosome 16p13.11 [6]. More than 450 pathogenic variants with variable phenotypic severities have been reported [7,8,9,10,11].

To the best of our knowledge, genetic analyses of Korean patients with PXE have not been reported to date. In the present study, we characterized the phenotypes of Korean patients with PXE and identified mutations in the *ABCC6* gene.

## 2. Materials and Methods

### 2.1. Design and Settings

This was a retrospective observational case series study. Patients visiting a single tertiary referral hospital and presenting AS in a fundoscopic examination were enrolled, between February 2014 and November 2020. The study involved a subset of patients included in a study of inherited retinal diseases (IRDs) approved by the Institutional Review Board (IRB) of the Seoul National University Bundang Hospital (IRB No. B-1105-127-014). It adhered to the tenets of the Declaration of Helsinki and was approved by the IRB of Seoul National University Bundang Hospital (IRB No. B-2012-655-102). Written informed consent was obtained from all participants.

### 2.2. Patient Evaluation

All patients underwent baseline ophthalmic examinations, including best-corrected visual acuity (BCVA) measurement, intraocular pressure measurement, dilated fundus examination, color fundus photography (VX-10a; KOWA, Tokyo, Japan), and spectral domain optical coherence tomography (SD-OCT; SPECTRALIS HRA+OCT, Heidelberg Engineering, Heidelberg, Germany). For a subset of patients, fluorescein angiography (FA) and indocyanine green angiography (ICGA) were conducted (SPECTRALIS HRA+OCT, Heidelberg Engineering). Fundus autofluorescence (FAF; SPECTRALIS HRA+OCT, Heidelberg Engineering) was performed in some cases. Ultra-widefield (UWF) fundoscopic images (200Tx, Optos, Dunfermline, Scotland) were obtained in five cases (#1, #2, #3, #5, and #6). Detailed patient data, including symptom onset, family history, and systemic diseases were gathered by a medical chart review [12].

### 2.3. Sample Collection and Sequencing

A customized target enrichment kit (Celemics, Seoul, Korea) was designed covering the exon and splicing regions of 254 genes related to IRDs. The captured library was sequenced using an illumina NextSeq550 instrument (Illumina, San Diego, CA, USA) to generate 2 × 150 bp reads. Alignment to the hg19 human genome (BWA-MEM), post-alignment and recalibration (‘Picard’ ver1.115 and ‘GATK’ ver4.0.4.0.), variant calling (GATK HaplotypeCaller), and annotation (ANNOVAR 2019Oct24) were performed. The Genome Aggregation Database (gnomAD; Cambridge, MA, USA), CADD, PolyPhen-2 and SIFT were used to exclude common variants and identify disease-causing variants. The assessment of variant pathogenicity followed the guidelines of the American College of Medical Genetics (ACMG).

### 2.4. Diagnosis of Pseudoxanthoma Elasticum

All patients were evaluated for PXE based on the criteria of Plomp et al. [2]. A definitive diagnosis was made when two of the three major criteria were met: skin involvement (pseudoxanthomas of neck/flexural areas or confirmed skin biopsy), eye involvement (peau d’orange or AS), and genetic confirmation (biallelic *ABCC6* variants or a first-degree relative). Probable PXE was diagnosed with the presence of two major eye or skin criteria or one major criterion and one or more minor criteria, including eye involvement with one AS shorter than one disc diameter or other ocular phenotypes (e.g., comets or wing signs) and genetic confirmation of a monoallelic *ABCC6* variant.

## 3. Results

Seven Korean patients with AS from unrelated families were enrolled. Demographic and clinical features are shown in Table 1. The median age at initial examination was 51.2 years (range: 32–69 years), and patients were followed up for a median of 3.2 years (range: 0.3–14.9 years). Four patients (#1, #2, #3, and #4) were asymptomatic and AS were seen at a routine fundus examination. Three patients (#4, #5, and #6) were symptomatic at presentation, with decreased visual acuity. The asymptomatic patients were significantly younger than the symptomatic patients (mean age; 38.1 vs. 61.5 years, *p* = 0.020) at presentation.

The median initial BCVA and BCVA at the last visit were 20/25 (range: 20/32 to 20/20) and 20/20 (range: 20/32 to 20/20) for asymptomatic patients, respectively. The median initial BCVA and BCVA at the last visit were 20/32 (range: 20/50 to 20/20) and 20/50 (range: 20/1000 to 20/32) for symptomatic patients, respectively.

Two patients (#1 and #4) had bilateral high myopia with a spherical equivalent of more than −6.0 diopters. Two subjects (#2 and #3) had a history of refractive surgery for myopia, however the refractive errors before the operation were unavailable. Five out of seven patients (71.4%) had skin involvement, and one patient (#6) was not checked for skin changes. Pathogenic *ABCC6* variants were found in all patients. According to the diagnostic criteria for PXE, definitive diagnoses were made in six patients (#1, #2, #4, #5, #6, and #7), and a probable diagnosis was made in one patient (#3).

Two patients (#5, and #7) had systemic hypertension, one patient (#5) had a history of paramedian pontine infarction, and one patient (#4) had a known cerebral aneurysm. The patients with systemic diseases underwent electrocardiography (ECG) and serum tests, including complete blood cell counts. ECG was performed in five patients (#1, #2, #5, #6, and #7); results revealed no abnormalities. Hemoglobin (Hb) levels were checked in all patients except for #3, and the results were within normal limits. The patients did not have symptoms related to renal and cardiovascular phenotypes or hemoglobinopathies other than known systemic diseases; thus, the patients were not referred to a cardiologist or a nephrologist.

### 3.1. Retinal Phenotypic Features of Patients

**Figure 1:**
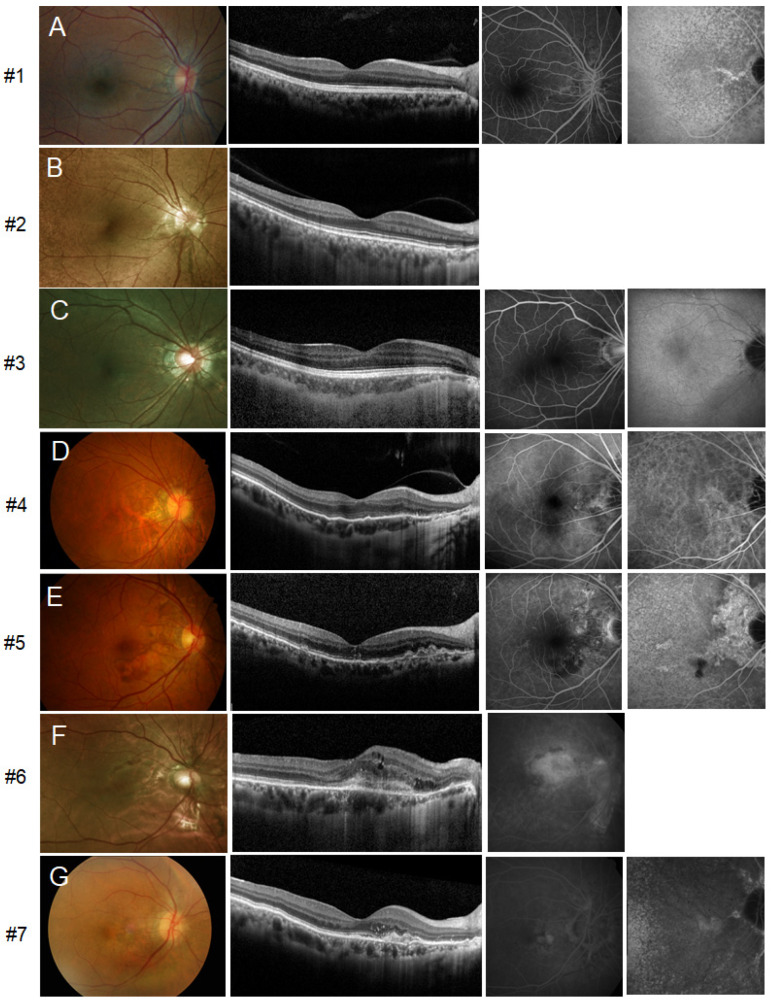
Multimodal imaging at the initial presentation. Images of the right eyes of patients are presented because both eyes exhibited symmetric features. From left to right, fundus photography, optical coherence tomography (OCT), fluorescein angiography (FA), and indocyanine green angiography (ICGA) are shown. (**A**–**D**) Fundus photographs of asymptomatic patients showing AS radiating from the optic disc. The horizontal OCT scan shows peripapillary disruption of the Bruch’s membrane-RPE complex and undulation of the overlying photoreceptor layers. AS was identified as hyperfluorescent lines without leakage on FA. Late-phase ICGA images showed a central zone of decreased fluorescence at the posterior pole, and AS showed an increased fluorescence signal within the central area of hypofluorescence. (**E**–**G**) Fundoscopy of right eye of patient #5 shows submacular hemorrhage and corresponding OCT reveals fibrovascular pigment epithelial detachment along with breakage of Bruch’s membrane. However, choroidal neovascularization (CNV) was not definitively identified with FA and ICGA, and the occurrence of spontaneous submacular hemorrhage due to Bruch’s membrane break is suspected (**E**). #6 had subfoveal CNV in the right eye, which was confirmed with FA (**F**). The initial presentation of #7 shows nasal parafoveal subretinal CNV in the right eye (**G**).

Initial multimodal imaging results for all patients with mild to severe retinal phenotypes are shown in Figure 1. Circumferential (360°) ‘peau d’orange’ centered on the posterior pole was visible, as assessed by UWF fundus photography (Figure S1). OCT revealed peripapillary disruption of the Bruch’s membrane-retinal pigment epithelium (RPE) complex, compatible with AS on fundoscopic examination. In FA images, AS showed hyperfluorescent lines without leakage. On late-phase ICGA images, decreased fluorescence centered on the posterior pole was noted, and AS showed a relatively increased fluorescence signal within a central zone of hypofluorescence. FAF revealed definite hypoautofluorescence around the disc extending to the fovea. One patient (#3) showed reticular pseudodrusen in both eyes. Other classic fundus findings such as optic nerve head drusen, comet-like peripheral crystalline bodies, or peripheral RPE atrophic spots were not present in our patients. Two symptomatic patients (#5 and #7) had unilateral submacular hemorrhage or CNV at the baseline examination (Figure 1E,G). Multimodal imaging of one patient (#6) revealed CNV in the subfoveal region of the right eye (Figure 1F) and nasal peripapillary area of the left eye.

**Figure 2:**
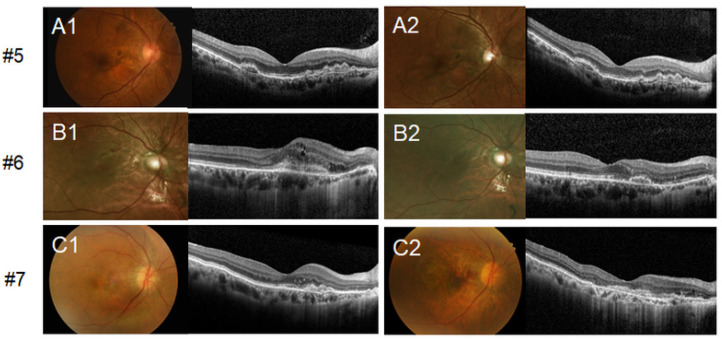
Changes in retinal phenotypes after the treatment of symptomatic patients (#5, #6, and #7). All symptomatic patients with choroidal neovascularization (CNV) were treated with intravitreal anti-vascular endothelial growth factor (anti-VEGF) injections. Only the right eyes of the patients are shown. Optical coherence tomography (OCT) of the right eye shows subretinal hyper-reflective tissue (**A1**–**C1**), which was confirmed as CNV by angiographic study. Reduction of subretinal CNV and absorption of subretinal fluid after intravitreal injections were noted (**A2**–**C2**). Patient #7 received 49 intravitreal injections in her right eye; subfoveal disciform scar with macular atrophy was observed consequently (**C2**).

All asymptomatic patients (#1, #2, #3, and #4) displayed baseline BCVA above 20/32 and maintained good visual acuity until the last follow-up visit without CNV occurrence. Three patients with initial visual loss (#5, #6, and #7) showed a deterioration of the BCVA and occurrence of CNV in both eyes during follow-up. A longitudinal evaluation of the retinal phenotype of asymptomatic patients (#1, #2, #3, and #4) demonstrated a slightly enlarged range of AS but without a definite interval change. Even in the patient with 15 years of follow-up (#1), only subtle thickening of streaks was noted. Symptomatic patients with initial vision loss (#5, #6, and #7) underwent treatment and the changes in the retinal phenotype before and after the treatment are presented in Figure 2. Patients were treated with intravitreal anti-vascular endothelial growth factor (anti-VEGF) injections, resulting in the reduction of subretinal CNV and absorption of the subretinal fluid. Patient #7 was initially diagnosed with neovascular age-related macular degeneration (AMD) and had experienced multiple recurrences of CNV during a follow-up period of 12 years. She received 49 intravitreal anti-VEGF injections in her right eye. Consequently, subfoveal disciform scar change and extensive macular atrophy occurred (Figure 2C2). Acute retinopathy in PXE, similar to multiple evanescent white dot syndrome, was not noted in any of the patients during follow-up [13].

### 3.2. Genotypes of Patients

Pathogenic *ABCC6* variants were found in all patients. Genetic testing revealed heterozygous monoallelic mutations in four patients (#1, #3, #4, and #5) and compound heterozygous mutations in three patients (#2, #6, and #7). None of the patients harbored a homozygous mutation. One variant, c.2542delA [p.Met848CysfsTer83], was detected in two patients (#3 and #7). In total, nine *ABCC6* variants were identified, and the data for these variants are summarized in Table 2. These included three nonsense mutations, three missense mutations, one frameshift, and a large deletion of exon 9 or deletion of the whole *ABCC6* gene. Among the variants, c.2035G>T [p.Glu679Ter] has not been previously reported. The other eight variants, including gross deletions, displayed low allele frequencies. All variants were pathogenic or likely pathogenic according to ACMG guidelines.

Only one patient (#7) had a male sibling with similar symptoms, however, genotyping of the family member could not be performed owing to his foreign residence. Four patients denied a family history of retinal diseases and a family history was not available for two patients. Therefore, the inheritance pattern of *ABCC6* could not be evaluated.

## 4. Discussion

We performed the first genotyping analysis of Korean patients with PXE and identified nine pathogenic variants of the *ABCC6* gene, including one novel variant. Three symptomatic patients with visual loss at initial presentation were aged older than 50 years and showed reductions in visual acuity and bilateral CNV occurrence during the follow-up, whereas four asymptomatic patients maintained good visual acuity without CNV occurrence. These patients with PXE showed late-onset clinical symptoms unlike those in other IRDs.

The *ABCC6* gene comprises 34 exons and encodes a transmembrane transporter protein primarily expressed in the liver and kidneys, with lower expression in the skin, eye, and vascular system [20,21]. The precise role of *ABCC6* is unknown; however, it is assumed to be associated with pyrophosphate metabolism. As pyrophosphate inhibits ectopic calcification, *ABCC6* mutations cause secondary calcification due to low pyrophosphate levels in the systemic circulation [22,23]. The phenotype of PXE is highly heterogenous among patients and even among family members carrying the same mutations [24]. The exact risk for cardiovascular system involvement in PXE remains unknown [2]. However, in an analysis of genotype-phenotype correlations in 289 patients with PXE, biallelic truncating variants were associated with more severe arterial and ophthalmological phenotypes than those of patients with mixed genotypes of one truncating and one non-truncating variant [3]. Additionally, severe skin phenotypes are correlated with cardiovascular and ophthalmic phenotypes [25,26].

The four asymptomatic patients (#1, #2, #3, and #4) were significantly younger than the symptomatic patients (#4, #5, and #6) at presentation, and three symptomatic patients were aged older than 50 years. Risseeuw et al. observed declining vision with increasing age, particularly in patients >50 years of age [27]. Gilem et al. also reported that the frequencies of macular atrophy and CNV increase with age [28]. Risseeuw et al. have also recently shown that the extent of AS increases with age and with the presence of CNV or macular atrophy in PXE [29].

The diagnosis of PXE can be delayed because the retinal phenotype of the disease mimics neovascular AMD. One of our patients (#7) with bilateral CNV at the age of 64 years was initially misdiagnosed with exudative AMD. Because PXE presents as bilateral ocular disease, careful regular retinal examinations of the opposite eye in patients with unilateral CNV may facilitate the early diagnosis and treatment of contralateral CNV and prevent severe bilateral visual loss. In our patients, CNV showed persistent recurrence without inactivation, resulting in visual loss at an earlier age than that observed with neovascular AMD [30]. In particular, Charbel Issa et al. reported three cases of the c.1171A>G variant in combination with a large deletion of *ABCC6* associated with mild retinal phenotypes of late-onset disease (80 years or later) and the absence of definite skin and cardiovascular changes. Late-onset PXE should be considered a differential diagnosis for AMD [7]. Birtel et al. found that OCT shows a higher diagnostic accuracy of CNV in PXE than OCT angiography and FA, especially with longitudinal follow-up images, and FA and OCT angiography might contribute to the diagnostic accuracy in more complex cases. The systematic use of OCT, OCT angiography, and FA may facilitate the diagnosis and monitoring of AS-related CNV in PXE [31]. Furthermore, in PXE, the transition from classic retinal dystrophies to systemic disease with a retinal phenotype could be indistinct and requires careful observations, as noted previously [1,12].

Charbel Issa et al. reported the topographic distribution of retinal phenotypes in patients with PXE by multimodal imaging and found three distinct areas centered on the posterior pole [32]. The central zone of decreased fluorescence in late-phase ICGA is highly characteristic finding in PXE. Eccentric to this central zone and first transition zone of normal fluorescence, the second transition zone, equivalent to peau d’orange, is fundoscopically visible, especially in darker-pigmented patients. The most peripheral area had a normal fundus reflex. We also observed a hypofluorescent central zone in late-phase ICGA and mid-peripheral circumferential (360°) area of peau d’orange in UWF fundoscopic images. These centrifugal positioning abnormalities in PXE may suggest underlying Bruch’s membrane calcification in PXE [32].

We observed diffuse reticular pseudodrusen in both eyes of one patient (#3). Gilem et al. previously described the association of reticular pseudodrusen with diseased Bruch’s membrane in PXE [33]. The reticular pseudodrusen of our patient was located central to the transitional zone of peau d’orange and anterior to the central macula, as described previously [33].

As PXE is an autosomal recessive disorder and the definitive diagnosis requires biallelic variants as a major criterion, heterozygous forms were detected in four of our patients (#1, #3, #4, and #5). Four patients with the monoallelic heterozygous mutation displayed a typical retinal phenotype. Three patients (#1, #3, and #4) were asymptomatic until the last follow-up; however, patient #5 with a novel truncation variant, c.2035G>T [p.Glu679Ter], experienced vision loss at presentation. Despite screening of all exons and splicing regions with adequate depth and checking for copy number variation, including large deletions, we did not find other *ABCC6* mutations. However, it is possible that a secondary variant, such as a deep intronic variant, was missed owing to the nature of the targeted gene panel. Recently, Gliem et al. observed a higher frequency of retinal alterations in monoallelic *ABCC6* carriers than in age-matched controls, indicating an association with late-onset haploinsufficiency [34]. Further studies including genetic testing of family members are needed to elucidate whether the genetic background is responsible for a higher susceptibility for a haploinsufficiency phenotype.

Furthermore, PXE-like lesions associated with hemoglobinopathies must be considered. These patients had acquired PXE-like lesions without *ABCC6* mutations [35]. The patients in the present study with heterozygous monoallelic *ABCC6* mutations did not have symptoms of hemoglobinopathies, and Hb levels were within normal limits.

The *ABCC6* frameshift variant (p.Met848CysfsTer8) in our cases (#3 and #7) was reported only in East Asian patients. All variants found in our study, except for p.Arg1314Trp, show high allele frequencies in East Asians. This indicates the presence of a shared genetic background among East Asian patients with PXE. There is an ethnic difference in the prevalence of *ABCC6* variants in European patients with PXE; the frequent variant of p.Arg1141Ter accounts for 1/3 of all mutations in this population [36]. Further studies with large sample sizes are required to identify ethnicity-specific variants in East Asians.

The present study had several limitations. First, selection bias could exist due to the small number of cases from a single center. Second, patients’ pedigrees could not be evaluated accurately; thus, the inheritance pattern might have been inaccurate. Furthermore, owing to the small number of cases, we could not evaluate the correlation between the progression of retinal phenotypes in PXE and structural measures such as subfoveal choroidal thickness. Lastly, as we utilized a targeted gene panel for IRDs, the library only contained the retinal genes and did not include other ectopic calcification genes, such as *GGCX*, *ENPP1*, *NT5E*, *SAMD9*, *FGF23*, and *MGP*.

## 5. Conclusions

In conclusion, we characterized the phenotypes of Korean patients with PXE, and identified *ABCC6* variants. Nine pathogenic variants of *ABCC6*, including one novel variant, c.2035G>T [p.Glu679Ter], were identified. The Korean patients with PXE and AS showed a late onset of clinical symptoms (>50 years of age). This is the first genetic analysis of *ABCC6* variants in Korean patients with PXE.

## Figures and Tables

**Table 1:** Demographic and clinical features of patients.

Patient ID	Sex	Age at Diagnosis (years)	Symptom	Follow-Up Duration (years)	BCVA at Baseline	BCVA at Last Follow-Up	Intravitreal Injections
#1	F	32	None	14.9	(OD) 20/20 (OS) 20/20	(OD) 20/20 (OS) 20/20	None
#2	F	37	None	3.2	(OD) 20/25 (OS) 20/25	(OD) 20/20 (OS) 20/20	None
#3	M	32	None	2.0	(OD) 20/20 (OS) 20/25	(OD) 20/20 (OS) 20/20	None
#4	F	51	None	0.3	(OD) 20/32 (OS) 20/25	(OD) 20/32 (OS) 20/25	None
#5	M	51	Sudden vision loss	3.9	(OD) 20/63 (OS) 20/20	(OD) 20/50 (OS) 20/100	(OD) Bevacizumab 5 times (OS) Bevaicuzumab 3 times
#6	F	64	Vision loss of unknown onset	0.6	(OD) 20/40 (OS) 20/25	(OD) 20/25 (OS) 20/32	(OD) Bevacizumab 4 times (OS) Bevaicuzumab 3 times
#7	F	69	Vision loss of unknown onset	12.1	(OD) 20/50 (OS) 20/25	(OD) 20/1000 (OS) 20/40	(OD) Bevacizumab 33 times, Ranibizumab 13 times, Aflibercept 3 times (OS) Bevacizumab 25 times

F = Female, M = Male, BCVA = Best-corrected visual acuity, OD = Oculus dextrus, OS = Oculus sinister.

**Table 2:** Profiles of variants in the *ABCC6* gene of patients.

Exon	Nucleotide Change	Protein Variant	Patient ID	CADD PHRED Score (GRCh37-v1.4)	Polyphen-2 (Score)	SIFT (Score)	MutationTaster	Allele Frequency (%) in gnomAD	Clinical Significance (ClinVar)	Pathogenicity (ACMG Classification)	Reference No.
9	c.1132C>T	p.Gln378Ter	#2	36	N/A	N/A	Disease causing	0.006	Pathogenic	Pathogenic	[14]
9	Del_Exon9	p.?	#2	N/A	N/A	N/A	N/A	N/A	N/A	Pathogenic	[15]
10	c.1256G>A	p.Arg419Gln	#6	23.9	Probably damaging (0.994)	Deleterious (0)	Disease causing	0.008	Pathogenic	Likely pathogenic	[16]
16	c.2035G>T	p.Glu679Ter	#5	48	N/A	N/A	Disease causing	None	Novel	Pathogenic	N/A (novel variant)
19	c.2419C>T	p.Arg807Trp	#6	29.3	Probably damaging (1.000)	Deleterious (0)	Disease causing	0.002	Pathogenic	Likely pathogenic	[17]
19	c.2542delA	p.Met848CysfsTer83	#3, #7	N/A	N/A	N/A	N/A	0.020	N/A	Pathogenic	[16]
28	c.3940C>T	p.Arg1314Trp	#4	32	Probably damaging (1.000)	Deleterious (0)	Disease causing	0.029	Pathogenic	Pathogenic	[6]
29	c.4192C>T	p.Arg1398Ter	#1	38	N/A	N/A	Disease causing	0.002	Pathogenic	Pathogenic	[18]
	Del_ABCC6		#7	N/A	N/A	N/A	N/A	N/A	N/A	Pathogenic	[19]

No. = Number, N/A = Not applicable, CADD (https://cadd.gs.washington.edu/score; accessed date: 26 April 2021), Polyphen-2 (http://genetics.bwh.harvard.edu/pph2; accessed date: 26 April 2021), SIFT (https://sift.bii.a-star.edu.sg; accessed date: 26 April 2021), MutationTaster (http://www.mutationtaster.org; accessed date: 26 April 2021), ACMG = American College of Medical Genetics and Genomics.

## Data Availability

The data presented in this study are available on request from the corresponding author. The data are not publicly available due to privacy restrictions concerning the study patients.

## Supplementary Materials

The following are available online at https://www.mdpi.com/article/10.3390/genes12081207/s1, Figure S1: Ultra-widefield fundoscopic photographs showing circumferential peau d’orange.
