# Supplementary material for: Clinical Characterization of Korean Patients with Pseudoxanthoma Elasticum and Angioid Streaks
Source: Genes (Basel). 2021 Aug 4;12(8):1207. doi: 10.3390/genes12081207 (PMC8391950; doi:10.3390/genes12081207)
Supplement: Supplementary file 1 [file genes-12-01207-s001.zip › genes-1313220-supplementary.pdf]

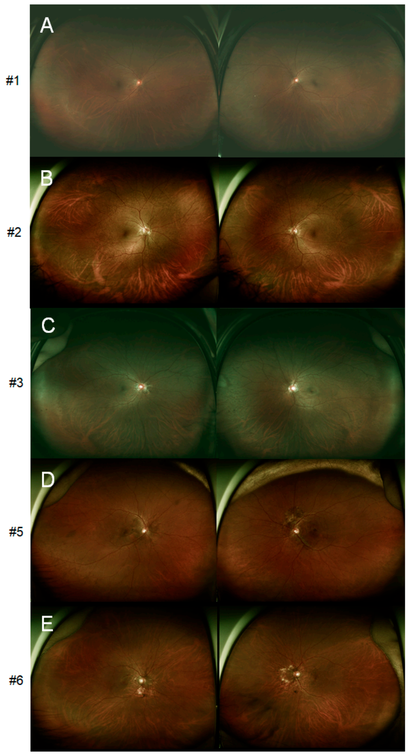

Figure S1. Ultra-widefield fundoscopic photographs showing circumferential peau d’orange.
Ultra-widefield fundoscopic images were obtained in five cases (#1, #2, #3, #5, and #6). Circumferential (360°) peau d'orange appearance centered on the posterior pole was visible, as previously reported as the second transition zone of centrifugal positioning abnormalities in pseudoxanthoma elasticum.
